# Climate Change and Extreme Weather Event Adaptations for Individuals With Dementia: A Systematic Review

**DOI:** 10.1002/brb3.71468

**Published:** 2026-05-11

**Authors:** Amanda Alvarez, Sidrah Rafiq, Catherine Chen

**Affiliations:** ^1^ Rutgers School of Graduate Studies Rutgers University Piscataway New Jersey USA; ^2^ Division of Geriatric Medicine, Department of Medicine, Robert Wood Johnson Medical School Rutgers University New Brunswick New Jersey USA; ^3^ Division of General Internal Medicine, Department of Medicine, Robert Wood Johnson Medical School Rutgers University New Brunswick New Jersey USA

**Keywords:** climate change, cognitive dysfunction, dementia, extreme weather

## Abstract

**Introduction:**

Dementia prevalence and the impacts of climate change are projected to rise significantly over the next three decades. Climate change vulnerability consists of three components: exposure, sensitivity, and adaptive capacity. While prior research has examined the exposure and sensitivity of persons with dementia, their adaptive capacity remains understudied. As climate change intensifies, understanding and enhancing the adaptive capabilities of persons with dementia is essential for developing climate‐resilient strategies. This review explores factors that contribute to the resilience of persons with dementia in the face of climate change and extreme weather events (EWEs).

**Methods:**

A systematic review of literature was conducted using Scopus, CINAHL, EMBASE, PubMed, ProQuest, and Web of Science databases, current as of March 2023. Included studies followed SPIDER guidelines and were published in English between January 2017 and February 2023.

**Results:**

Of 473 screened articles, 13 met the inclusion criteria: 5 cohort studies, 4 qualitative studies, 3 cross‐sectional studies, and 1 prevalence study. Quality assessment was conducted using the JBI SUMARI Critical Appraisal Tool and data was synthesized narratively. Three key themes emerged: (1) effects of climate conditions on cognitive function, (2) importance of socialization, and (3) standards for best practice.

**Conclusion:**

Socialization and community support are critical in preserving cognitive function and enhancing the adaptive capacity of persons with dementia in response to EWEs. To improve outcomes, specialized training for caregivers, shelter staff, and emergency responders is necessary. Policies must also strengthen support for people with dementia and their caregivers. Future research should explore the needs of individuals at different stages of dementia and develop targeted interventions to foster resilience and protect their well‐being in emergencies. PROSPERO ID: CRD42023414468

## Graphical abstract

This systematic review of 13 studies identifies factors for the adaptive capacity of persons with dementia during extreme weather events. Key findings indicate that while social isolation and displacement exacerbate cognitive decline, community support and socialization may enhance resilience. The review calls for specialized training for emergency responders and caregivers, alongside policy improvements, to foster climate‐resilient care models.

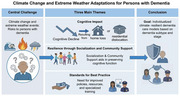

**Abbreviations**
ADRD: Alzheimer's disease and related dementias
EWE: extreme weather events
GEJET: Great East Japan Earthquake and Tsunami
PWCD: Persons with cognitive decline
PWCD: Persons with cognitive decline
PWCI: persons with cognitive impairment
SES: socioeconomic status

## 1 Introduction

Climate change poses an imminent threat to the balance of our ecosystems and to the well‐being of humanity (Wei et al. 2019). The Intergovernmental Panel on Climate Change (IPCC) 2023 assessment report states that risks and associated losses or damages from climate change will intensify with every increment of global warming (Calvin et al. 2023).

In 2020, there were over 55 million people worldwide who had dementia, with numbers projected to triple to 152 million by 2050 (Y. Zhang et al. 2023). According to the World Health Organization (WHO), dementia is the seventh leading cause of death and one of the main causes of disability and dependency among the global population of older adults (World Health Organization 2018; Toots et al. 2016).

The heightened frequency and intensity of extreme weather events (EWEs) associated with climate change, such as hurricanes, floods, and heat waves, can displace people with dementia from their familiar environments and support systems during disasters (Dosa et al. 2023). Evacuations and emergencies can be disorienting for persons with dementia as they may wander, lack insight into the nature of the emergency, and struggle to follow complex evacuation procedures (Dosa et al. 2023). Disruption of routines can trigger confusion and exacerbate the emotional and psychological toll on persons with dementia, reducing compliance with evacuation efforts, especially in individuals with early to middle stages of dementia (Dosa et al. 2023; Christensen and Castañeda 2014).

After natural disasters, the rebuilding process and changes in living conditions can complicate matters for people with dementia (Akerlof et al. 2015). Disrupted healthcare infrastructures may hinder access to essential medical support, while loss of belongings or relocation to unfamiliar settings can heighten stress and disorientation (Gong et al. 2022). Caregivers, often family members, face increased challenges managing the complex needs of persons with dementia in the aftermath of disasters (Dawson et al. 2019). While prior research has addressed the relationship between climate change and vulnerabilities of this demographic, there remains a gap in understanding the adaptive capacities of persons with dementia for mitigating these effects (Gaskin et al. 2017).

Addressing the barriers that hinder people with dementia's functionality will ensure their safety, well‐being, and quality of life amid changing climates (Dosa et al. 2023; Berghs 2020). This systematic review aims to identify factors contributing to persons with dementia adaptive capacity regarding climate change and EWEs.

## 2 Methods

### 2.1 Search Strategy

The protocol for the systematic review was registered with PROSPERO in April 2023 (ID: CRD42023414468) and conducted following PRISMA guidelines (Page et al. 2021). A literature search was performed (November 2022–March 2023) across six electronic databases: Scopus, CINAHL, EMBASE, PubMed, ProQuest, and Web of Science. A list of terms and tailored Boolean operators for each database is in Table 1.

**TABLE 1:** Search strategy per database.

Database	Search strategy
CINAHL	((Climate change) OR (natural disaster) OR (extreme weather)) AND (dementia OR (cognitive dysfunction))
EMBASE	(‘climate change’/exp OR ‘climate change’ OR ‘natural disaster’/exp OR ‘natural disaster’ OR ‘extreme weather’/exp OR ‘extreme weather’) AND ((‘dementia’/exp OR ‘dementia’) OR ‘cognitive dysfunction’/exp OR ‘cognitive dysfunction’)
ProQuest	noft(climate change OR natural disaster OR extreme weather) AND noft(dementia OR cognitive dysfunction)
PubMed	(((((“Climate Change”[Mesh]) OR (“Natural Disasters”[Mesh])) OR (“Extreme Weather”[Mesh])) OR (climate change [tiab])) OR (extreme weather [tiab] OR natural disaster [tiab])) AND ((((((“Dementia”[Mesh]) OR (“Cognitive Dysfunction”[Mesh]))) OR (dementia [tiab])) OR (cognitive dysfunction [tiab])))
Scopus	TITLE‐ABS (({climate change} OR {natural disaster} OR {extreme weather}) AND (dementia OR {cognitive dysfunction}))
Web of Science	(ALL = (climate change) OR ALL = (natural disaster) OR ALL = (extreme weather)) AND (ALL = (dementia) OR ALL = (cognitive dysfunction))

### 2.2 Eligibility Criteria

Studies were selected based on SPIDER guidelines (Table 2). Studies included in this review met the following criteria: (1) persons with dementia are the participants or data is provided on this population (studies that did not explicitly discuss persons with dementia but defined the participants level of cognitive decline were included), (2) exposures were climate change‐related events (e.g., droughts, floods, heat waves, hurricanes, and wildfires), (3) publication period was from January 2017 to February 2023, (4) focus on factors influencing the adaptive capacity of persons with dementia, including but not limited to personal and environmental factors (e.g., support from family, friends, caregivers), activities, and participation, and (5) qualitative, quantitative, and mixed methods studies were eligible. No geographic restrictions were applied; only English‐language publications were included. Articles focusing solely on persons with dementia without discussing exposures related to climate change, and vice versa, were excluded.

**TABLE 2:** Inclusion criteria for the systematic review using SPIDER (sample, phenomenon of interest, design, evaluation, and research type) framework.

Framework	Eligibility criteria
Sample	Persons with dementia are the participants or data is provided on this population (studies that provided data for persons with cognitive dysfunction [PWCD] were only included if they defined the status of cognitive dysfunction of the participants)
Phenomenon of interest	The exposures are events that are likely to be associated with climate change (e.g., droughts, floods, heat waves, hurricanes, and wildfires)
Design	The publication period of the searches for this review was from January 2017 to February 2023, no geographic regions were excluded, and the search was limited to English‐language publications
Evaluation	Factors contributing to the adaptive capacity of persons with dementia are identified, including but not limited to personal factors, environmental factors (e.g., support from family, friends, caregivers), activities and participation
Research type	Qualitative, quantitative, and mixed methods studies

### 2.3 Study Selection Process

Articles were imported into Rayyan (Ouzzani et al. 2016) and underwent title and abstract screening by two independent reviewers, A.A. and C.C. Articles were excluded at the title/abstract screening stage if they: (1) did not discuss persons with dementia, (2) did not discuss climate‐change related exposures, (3) articles were outside the publication date range (January 2017–February 2023), and (4) article was not written in English. Full‐text screening applied these criteria plus: (1) ineligible study design, and (2) no discussion of the adaptive capacities of persons with dementia in response to climate weather events. Disagreements during the study selection process were resolved by discussion between A.A. and C.C.

### 2.4 Data Extraction and Analysis

A.A. and C.C. independently assessed study quality using the JBI SUMARI Critical Appraisal Tool (JBI n.d.) with appropriate checklists for each article type. Extracted data (e.g., authors, year, methods, country of origin, phenomena of interest, setting, participant details, key findings) were coded, consolidated in Microsoft Excel and synthesized narratively.

Themes were identified inductively from cohort, qualitative, cross‐sectional, and prevalence studies as reported by the original authors if available or during coding by A.A. and C.C. Original study themes were categorized and refined through discussion until both authors reached a consensus.

## 3 Results

**FIGURE 1:**
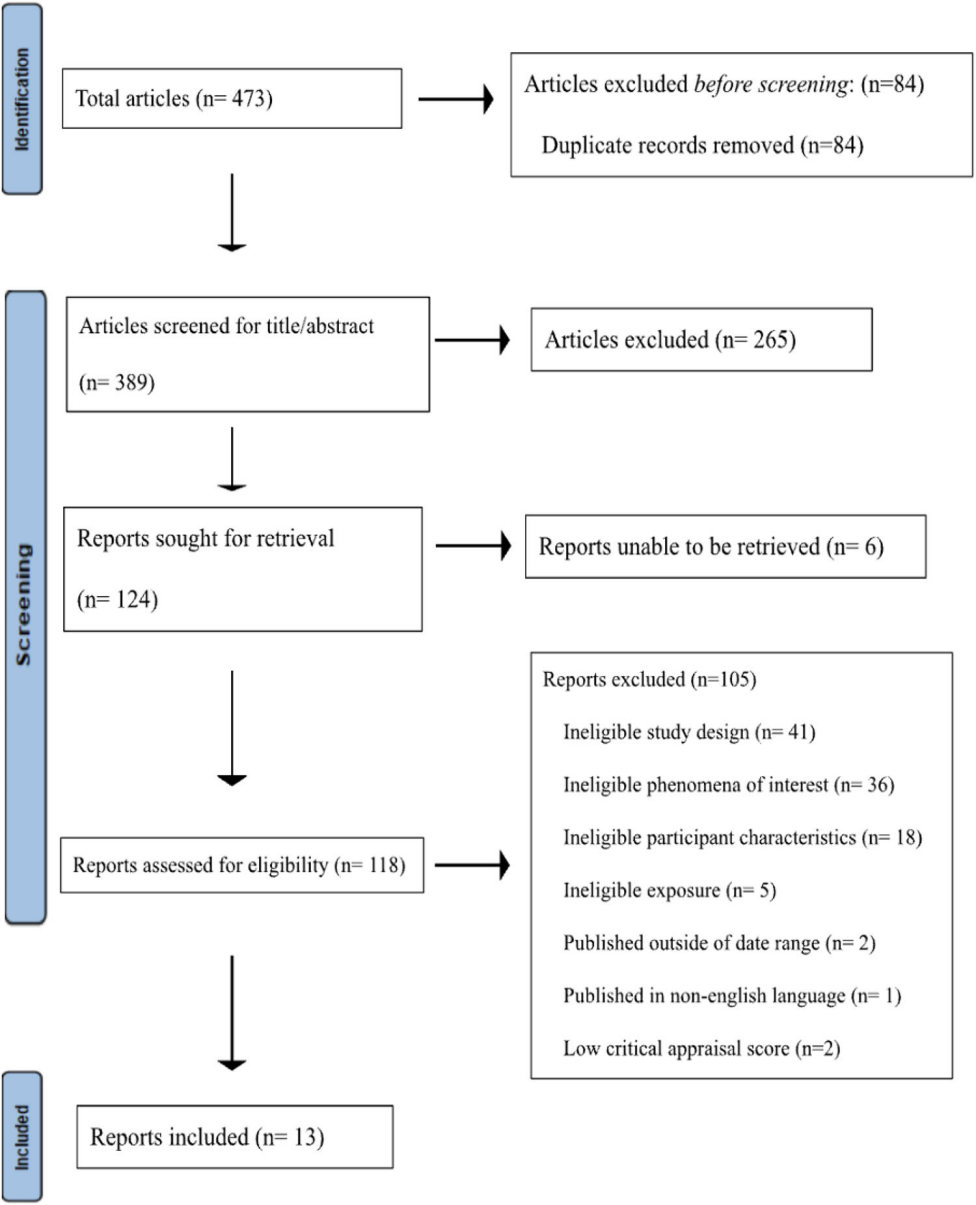
PRISMA flowchart: identification and selection of studies for the systematic review.

From the literature search, 473 references were retrieved. Eighty four duplicates were removed before the screening process began. Out of 389 articles that underwent title/abstract screening, 265 articles were excluded. Of 124 full‐text articles sought, 6 could not be retrieved. The remaining 118 articles underwent full‐text screening. A total of 103 articles were excluded. The detailed search and selection process is outlined in the PRISMA flowchart (Figure 1). Cohen's kappa value (0.363) from the full text screening showed fair agreement between the two reviewers, A.A. and C.C. (Landis and Koch 1977).

### 3.1 Risk of Bias Assessment

Of the 15 remaining articles, two with quality assessment scores below 50% were deemed to have a high risk of bias and excluded during the critical appraisal stage. Eleven studies had an overall low risk of bias (100%–80%), and two had a moderate risk of bias (79%–60%) (Barker et al. 2023). The JBI SUMARI Critical Appraisal results are detailed in Table 3.

**TABLE 3:** JBI SUMARI critical appraisal responses and inclusion determined by percentages.

Type of study	Authors, year	Question (Q) 1	Q 2	Q 3	Q 4	Q 5	Q 6	Q 7	Q 8	Q 9	Q 10	Q 11	%
Analytical cross sectional	Gonzalez Ramirez et al. (2019)	N	U	N	Y	N	N	Y	U				25
Analytical cross sectional	Hikichi et al. (2020)	Y	Y	Y	Y	Y	Y	Y	Y				100
Analytical cross sectional	Schnitker et al. (2019)	U	Y	Y	Y	Y	Y	Y	Y				87.5
Analytical cross sectional	Woolford et al. (2022)	Y	Y	Y	Y	Y	U	Y	Y				87.5
Cohort	Hikichi et al. (2019)	N/A	N/A	Y	Y	Y	Y	Y	Y	Y	Y	Y	81.8
Cohort	Finlay et al. (2020)	N/A	N/A	Y	Y	Y	Y	Y	Y	Y	U	Y	72.7
Cohort	Miyamori et al. (2023)	Y	Y	Y	Y	Y	Y	Y	Y	N	N	Y	81.8
Cohort	Shiba et al. (2021)	Y	Y	Y	Y	Y	Y	Y	Y	Y	Y	Y	100
Cohort	Yoshida et al. (2021)	Y	Y	Y	Y	Y	U	Y	Y	U	N	Y	72.7
Prevalence	Parks et al. (2021)	Y	Y	Y	Y	Y	Y	Y	Y	N/A			88.8
Qualitative	Boucher et al. (2022)	Y	Y	Y	Y	Y	N	Y	Y	Y	Y		90
Qualitative	Gibson et al. (2018)	Y	Y	Y	Y	Y	N	Y	Y	Y	Y		90
Qualitative	Nicosia et al. (2022)	Y	Y	Y	Y	Y	Y	Y	Y	Y	Y		100
Qualitative	Uekusa (2019)	Y	Y	Y	Y	Y	Y	Y	Y	Y	Y		100
Systematic review and research syntheses	Sepulveda et al. (2018)	Y	N	Y	U	N	U	N	N	N	N	N	18.1

*Note*: Analytical cross‐sectional questions: Q1. Were the criteria for inclusion in the sample clearly defined? Q2. Were the study subjects and the setting described in detail? Q3. Was the exposure measured in a valid and reliable way? Q4. Were objective, standard criteria used for measurement of the condition? Q5. Were confounding factors identified? Q6. Were strategies to deal with confounding factors stated? Q7. Were the outcomes measured in a valid and reliable way? Q8. Was appropriate statistical analysis used?

Cohort questions: Q1. Were the two groups similar and recruited from the same population? Q2. Were the exposures measured similarly to assign people to both exposed and unexposed groups? Q3. Was the exposure measured in a valid and reliable way? Q4. Were confounding factors identified? Q5. Were strategies to deal with confounding factors stated? Q6. Were the groups/ participants free of the outcome at the start of the study (or at the moment of exposure)? Q7. Were the outcomes measured in a valid and reliable way? Q8. Was the follow up time reported and sufficient to be long enough for outcomes to occur? Q9. Was follow up complete, and if not, were the reasons to loss to follow up described and explored? Q10. Were strategies to address incomplete follow up utilized? Q11. Was appropriate statistical analysis used?

Prevalence questions: Q1. Was the sample frame appropriate to address the target population? Q2. Were the study participants sampled in an appropriate way? Q3. Was the sample size adequate? Q4. Were the study subjects and the setting described in detail? Q5. Was the data analysis conducted with sufficient coverage of the identified sample? Q6. Were valid methods used for the identification of the condition? Q7. Was the condition measured in a standard, reliable way for all participants? Q8. Was there appropriate statistical analysis? Q9. Was the response rate adequate, and if not, was the low response rate managed appropriately?

Qualitative questions: Q1. Is there congruity between the stated philosophical perspective and the research methodology? Q2. Is there congruity between the research methodology and the research question or objectives? Q3. Is there congruity between the research methodology and the methods used to collect data? Q4. Is there congruity between the research methodology and the representation and analysis of data? Q5. Is there congruity between the research methodology and the interpretation of results? Q6. Is there a statement locating the researcher culturally or theoretically? Q7. Is the influence of the researcher on the research, and vice‐ versa, addressed? Q8. Are participants, and their voices, adequately represented? Q9. Is the research ethical according to current criteria or, for recent studies, and is there evidence of ethical approval by an appropriate body? Q10. Do the conclusions drawn in the research report flow from the analysis, or interpretation, of the data?

Systematic review and research syntheses questions: Q1. Is the review question clearly and explicitly stated? Q2. Were the inclusion criteria appropriate for the review question? Q3. Was the research strategy appropriate? Q4. Were the sources and resources used to search for studies adequate? Q5. Were the criteria for appraising studies appropriate? Q6. Was critical appraisal conducted by two or more reviewers independently? Q7. Were the methods to minimize errors in data extraction? Q8. Were the methods used to combine studies appropriate? Q9. Was the likelihood of publication bias assessed? Q10. Were recommendations for policy and/or practice supported by the reported data? Q11. Were the specific directives for new research appropriate?

Abbreviations: Y‐ Yes, N‐ No, U‐ Unclear, N/A‐ Not applicable.

### 3.2 Clarification of Terms

The DSM‐V defines mild cognitive impairment (MCI), also known as mild neurocognitive disorder, as a deficit in one of six cognitive domains, with individuals able to function independently with some accommodations (Sachs‐Ericsson and Blazer 2015; Hugo and Ganguli 2014). Dementia, or major neurocognitive disorder, is defined as MCI with impairments in activities of daily living (ADL) or instrumental activities of daily living (IADL) and there exists a high degree of functional variation among persons with dementia (Sachs‐Ericsson and Blazer 2015; Hugo and Ganguli 2014). Many individuals on the cusp of dementia can maintain functionality within structured environments, performing tasks without extensive resources, time, or caregiving support (Gale et al. 2018).

In the reviewed articles, participants are described as having Alzheimer's disease and related dementias (ADRD), as having dementia staged using scales for inclusion, or as caregivers of persons with dementia. Five articles categorized participants as persons with cognitive impairment (PWCI) (Hikichi et al. 2020; Shiba et al. 2021; Miyamori et al. 2023; Hikichi et al. 2019; Nicosia et al. 2022), while one article used the term persons with cognitive decline (PWCD) for MCI cases without ADL/IADL deficiencies (Finlay et al. 2020). To maintain consistency, all participants will be referred to as people with dementia, except when distinguishing between PWCD and PWCI.

### 3.3 Study Characteristics

**FIGURE 2:**
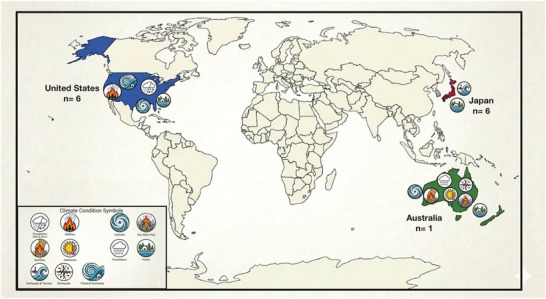
Geographic distribution of included studies by climate condition. Map and climate condition symbol legend created separately using Gemini 3 Flash Image. Both images were combined and modified using Goodnotes by author A.A., with the final image cross‐checked against Table 4 and reviewed for accuracy by all authors.

The final review included 13 studies on adaptations for persons with dementia associated with climate change and EWEs. Six studies originated from Japan, six from the United States, and one from Australia (Figure 2). Among the US studies, three collected nationwide data, two focused on the southeast/Gulf Coast states, and one was specific to California (Table 4).

**TABLE 4:** Summary of included literature and themes identified, categorized by climate condition.

Geographic location, study, year	Phenomena of interest	Methods and participants	Climate condition	Type of dementia/dementia status	Themes identified
**Earthquake and tsunami**					
**Effects on cognitive function**: Displacement and disruption to living environments were consistently associated with cognitive decline **Socialization**: Loss of social networks and community structures contributed to increased isolation and reduced resilience **Best practices related to non‐evacuation/ongoing support**: Continuity of care and environmental familiarity emerged as key protective factors **Best practices related to evacuation**: Evacuation and relocation processes introduced significant stress, requiring structured support and planning					
Japan, Hikichi et al. (2019)	Explore the long‐term influence of housing damage/residential relocation on cognitive disability of older adult survivors of the Great East Japan Earthquake and Tsunami (GEJET)	Database: Surveying used to compile database; *n* = 5058 residents aged 65+ at baseline, *n* = 2760 at 5.5‐year follow‐up	Earthquake and tsunami	Type of dementia not reported; level of cognitive deficit was outlined	Effects of climate conditions on cognitive function; importance of socialization
Japan, Hikichi et al. (2020)	To prospectively examine the association between changes in neighborhood social capital and cognitive function, spanning the experience of disaster	Survey; *n* = 5058 residents aged 65+ at baseline, *n* = 3350 at 2.5‐year follow‐up	Earthquake and tsunami	Type of dementia not reported; level of cognitive decline was outlined	Effects of climate conditions on cognitive function; importance of socialization
Japan, Shiba et al. (2021)	To examine the association of two types of traumatic events and the level of cognitive disability among older adult survivors of the GEJET	Database; *n* = 3350 residents aged 65+, in category one→ home loss = 148 vs. no home loss or less severe damage = 3112, in category two→ loss of a loved one = 1254 vs. no loss of loved ones = 2096	Earthquake and tsunami	Type of dementia not reported; level of cognitive deficit was outlined	Effects of climate conditions on cognitive function; importance of socialization
Japan, Uekusa (2019)	Explore the experiences of caregivers who provided support to elderly sufferers of AD and other related forms of dementia during the 2011 GEJET	Interview; *n* = 8 caregivers, all eight caregivers were caring for persons with dementia, five of which were female	Earthquake and tsunami	AD/ADRD; dementia status not reported	Updating standards of best practice
**Floods and tropical cyclones**					
**Effects on cognitive function**: Both immediate impacts and prolonged disruption to care and infrastructure contributed to cognitive and functional decline **Socialization**: Caregiver burden and social isolation were consistently reported across studies **Best practices related to non‐evacuation/ongoing support**: Effective response strategies emphasized coordinated support systems and clear communication **Best practices related to evacuation**: Recurrent evacuation scenarios highlighted the need for dementia‐capable shelters and caregiver‐inclusive planning					
Australia, Schnitker et al. (2019)	Explore the natural disaster strategies of Australian residential aged care facilities (RACF's), focusing on aspects relevant to persons with dementia	Survey and thematic analysis; *n* = 416 respondents, 321 of which were managers/ directors of nursing homes;	Floods, bushfires, cyclones, precipitation (superstorm rain and hail), heatwaves, earthquake, (non‐bush) fires	Type of dementia and dementia status not reported; Mean percentage of residents with dementia = 48%	Updating standards of best practice
Japan, Miyamori et al. (2023)	To analyze how the 2018 Japan floods impacted nursing home admissions for older persons using the Long‐Term Care Insurance (LTCI) database	Database; *n* = 188,589 LTCI users aged 65+, group one *n* = 2156, group two *n* = 185,705	Floods	Type of dementia not reported; level of cognition was outlined	Effects of climate conditions on cognitive function; importance of socialization; updating standards of best practice
Japan, Yoshida et al. (2021)	To reveal the effect of the 2018 Japan floods on the cognitive decline of vulnerable elderly	Database; *n* = 264,614 LTCI users aged 65+, group one *n* = 2908, group two *n* = 261,671	Floods	Type of dementia not reported; level of dementia was outlined	Importance of socialization; updating standards of best practice
United States, Parks et al. (2021)	Evaluate how tropical cyclone exposure is associated with increased hospitalization rates in older adults	Database; *n* = 69,682,674 Medicare hospitalizations of residents aged 65+ over a 16‐year study period	Cyclones	Type of dementia and dementia status not reported	Effects of climate conditions on cognitive function; updating standards of best practice
Southeast/Gulf Coast, United States, Boucher et al. (2022)	To ascertain common experiences and needs of a diverse group of caregivers challenged by hurricanes/floods and COVID‐19	Interview and thematic analysis; *n* = 25 caregivers, 20 of which were female, 15 were spousal and 21 lived with recipient	Floods and hurricanes	Type of dementia not reported; dementia status not reported	Updating standards of best practice
South Carolina, United States, Gibson et al. (2018)	Explore the experience of caregivers of persons with ADRD during the South Carolina flooding and subsequent recovery	Interview and thematic analysis; *n* = 27 caregivers, 24 of which were female, 21 were spousal and 25 lived with recipient	Floods	AD/ADRD; dementia status not reported	Importance of socialization; updating standards of best practice
**Precipitation**					
**Effects on cognitive function**: Environmental conditions influenced cognitive outcomes indirectly through effects on mobility, activity, and stress **Socialization**: Reduced social engagement and increased indoor confinement were linked to cognitive decline **Best practices related to non‐evacuation/ongoing support**: Maintaining routine, physical activity, and social interactions emerged as key protective strategies **Best practices related to evacuation**: Adaptation strategies focused primarily on monitoring and supporting individuals in place rather than evacuation					
United States, Finlay et al. (2020)	Examination of the relationship between annual exposure to precipitation and age‐based trajectories of cognitive function of older Americans	Interview and cognitive assessment; *n* = 25,320 residents aged 64+	Precipitation (rain and snow)	Type of dementia not reported; level of cognition was outlined	Importance of socialization; updating standards of best practice
United States, Woolford et al. (2022)	To examine the factors that were associated with being found alive compared to being found dead in search and rescue incidents of persons 65 years of age and older in the United States	Database; *n* = 1703 individuals of search and rescue incidents aged 65+, in Group 1 *n* = 692/1495 had dementia, in Group 2 *n* = 53/208 had dementia	Precipitation (rain and snow)	Type of dementia not reported; Status: Group 1 Severe dementia = 48.6% Moderate impairment = 44.5% Group 2 Moderate impairment = 87.4%	Updating standards of best practice
**Wildfires**					
**Effects on cognitive function**: Disruption to routines and environments contributed to increased stress and cognitive vulnerability **Socialization**: Caregiver strain was a consistent factor influencing outcomes **Best practices related to non‐evacuation/ongoing support**: Engagement in structured activities and support programs supported resilience **Best practices related to evacuation**: Evacuation‐related stress highlighted the importance of preparation and behavioral support strategies					
California, United States, Nicosia et al. (2022)	To explore the effects of the northern California wildfires on caregivers perceived stress and cognitive function, physical function, and quality of life among PWCD	Survey; *n* = 17 PWCD/caregiver dyads, 13 of which returned at least one survey, 9 returned both surveys and all 13 respondents were female	Wildfires	Type: 31% = Alzheimer's 38% = unspecified dementia 31% = other diagnoses Status: 46% = moderate dementia (clinical dementia rating: 2) 31% = mild dementia (clinical dementia rating: 1) 23% = mild cognitive impairment (clinical dementia rating: 0.5)	Importance of socialization

Participants included residents of the study location aged 64+ (*n* = 8), caregivers of persons with dementia (*n* = 3), nursing home managers (*n* = 1), and PWCI/caregiver dyads (*n* = 1). Most caregivers were female, spousal partners, and lived with the care recipient (Parks et al. 2021; Yoshida et al. 2021; Gibson et al. 2018). Two studies specified the participants had higher educational attainment, while one focused on participants from low socioeconomic status (SES) backgrounds (lower household incomes and fewer years of schooling), particularly those who experienced home loss due to EWEs (Shiba et al. 2021; Nicosia et al. 2022; Boucher et al. 2022).

Climate exposures included floods/hurricanes (*n* = 4), earthquakes/tsunamis (*n* = 4), precipitation (*n* = 2), cyclones (*n* = 1), wildfires (*n* = 1), and multiple climate conditions (*n* = 1). These exposures reflect both acute EWEs and broader environmental conditions, which were not consistently distinguished across studies. Findings were synthesized thematically, with patterns identified across studies and further examined in relation to event type and geographic context (Table 4). Three studies specified Alzheimer's disease/ADRD as the dementia type, while 10 did not report the dementia type. Two studies reported the degree of impairment of the participants, six defined cognitive function/deficits, and five did not specify dementia status.

### 3.4 Common Themes Identified

Three common themes were identified including (1) the effects of climate conditions on cognitive function, (2) the effects of isolation and the importance of socialization, and (3) standards for best practice.

#### 3.4.1 The effects of climate conditions on cognitive function

Five articles discussed how the destruction of homes and loss of loved ones due to EWEs exacerbate cognitive decline in persons with dementia and PWCI. Three articles used data from a 6‐year longitudinal study of individuals aged 65+ affected by the 2011 Great East Japan Earthquake and Tsunami (GEJET). Home loss was associated with increased cognitive disability 2.5 years after the disaster and a sustained linear exposure‐response relationship after 5.5 years (Hikichi et al. 2020; Shiba et al. 2021; Hikichi et al. 2019).

Greater housing damage was associated with greater cognitive decline. People whose homes were destroyed were moved into temporary housing villages, similar to Federal Emergency Management Agency (FEMA) housing in the United States (Hikichi et al. 2020; Hikichi et al. 2019). After 5.5 years, temporary housing closed, forcing residents to move into privately owned or government‐provided housing with highest risk of decline in the latter (Hikichi et al. 2019). Given the small sample of 31 individuals, the researchers suggest this was due to cognitively vulnerable individuals being more likely to have opted to move into rental housing rather than purchase a new home after the temporary housing closures (Hikichi et al. 2019).

Two studies linked long‐term disaster impacts to the stress of property damage or destruction (Hikichi et al. 2019; Parks et al. 2021). Researchers suggested that residential dislocation and resulting social isolation were key drivers of cognitive decline in cognitively frail individuals rather than psychological trauma from housing destruction (Hikichi et al. 2020; Shiba et al. 2021). EWEs also impacted ADLs, specifically eating, sleeping, and toileting, due to fragmented infrastructure (Miyamori et al. 2023).

#### 3.4.2 The effects of isolation and the importance of socialization

Eight articles addressed this theme. Four examined how social isolation contributes to cognitive decline in older adults, with factors like living alone exacerbating the issue in the context of variable effects of annual precipitation (Shiba et al. 2021; Finlay et al. 2020). Six highlighted the importance of social engagement and community support for preserving cognitive function and resilience in persons with dementia after EWEs.

1. *Effects of isolation on cognition*: Old age (80+ years), unmarried status (widowed, divorced, single), and living alone increased social isolation among older adult survivors in the GEJET study (Shiba et al. 2021). Social isolation, combined with limited mobility, physical stress, and reduced mental/social activity, contributed to cognitive decline (Miyamori et al. 2023). Participants in lower‐population‐density areas had higher cognitive decline rates (Yoshida et al. 2021).
  One article compared the effect of precipitation in five Norwegian communities on cognitive decline in adults aged 45 and older (Finlay et al. 2020). Higher cognitive function and slower initial decline was seen in adults exposed to moderate annual precipitation (20%–40% of days per year) than those exposed to infrequent precipitation (< 20% of days) (Finlay et al. 2020). Cognitive function in both groups converged by age 70 (Finlay et al. 2020). Moderate precipitation may reduce high temperatures, encouraging physical activity and social engagement (Finlay et al. 2020). Very high frequency of precipitation (50%–66% of days) was associated with the fastest rates of cognitive decline due potentially to indoor confinement from hazards of rain, snow, and ice leading to elevated stress levels and reduced mental stimulation (Finlay et al. 2020).
2. *Socialization and the adaptive potential of persons with dementia*: Involvement in community‐based activity centers and light physical activities helps preserve cognitive function in older adults (Hikichi et al. 2019). One article described a study of a non‐pharmacological activity‐based intervention called Preventing Loss of Independence through Exercise (PLIE) for people with cognitive impairment (Nicosia et al. 2022). They observed resilience and increased self‐efficacy in some caregivers and improved adaptability and cognitive function in people with cognitive impairment following the 2017 California wildfires (Nicosia et al. 2022).
  Three articles emphasized that social connections are crucial for resilience following natural disasters, suggesting that recovery relies more on social bonds than rebuilding physical infrastructure (Hikichi et al. 2020; Shiba et al. 2021; Hikichi et al. 2019). Participation in social activities, community participation, and informal socializing helped mitigate the effects of housing damage and residential dislocation on cognitive decline (Hikichi et al. 2020; Shiba et al. 2021; Hikichi et al. 2019). Refugees in shelters experienced difficulties in maintaining social activities and dealing with mental distress from losing family members (Yoshida et al. 2021).
  Participation in assistance programs or adult day centers improved recovery outcomes by providing continuity and support during tragedy (Hikichi et al. 2020; Shiba et al. 2021). Behavioral improvements were noted when residents of one community facility relocated together (Nicosia et al. 2022).

#### 3.4.3 Standards for Best Practice

Nine articles suggested improving the standards of best practice by enhancing disaster preparedness and relief efforts to address the specific needs of persons with dementia and their caregivers (Gibson et al. 2018). This should involve more flexible and targeted interprofessional services such as education, advocacy, research, and social worker involvement (Miyamori et al. 2023). Recommendations varied depending on whether evacuation or non‐evacuation strategies and ongoing support were required.

1. *Best practices related to evacuation*: Caregivers reported challenges balancing caregiving and disaster response, delaying decision‐making (Gibson et al. 2018). They expressed anxiety about disrupted medical services and noted that standard shelters were unsuitable for care recipients who could not handle excess stimulation (Boucher et al. 2022). Public shelters posed issues like general discomfort, lack of privacy, and shelter workers' lack of understanding of dementia‐related behaviors (Yoshida et al. 2021; Gibson et al. 2018). Training shelter employees to identify cognitive impairment during initial screenings and accommodate the needs of people with dementia suggested (Gibson et al. 2018).
  Recommendations included creating shelters for older or incapacitated individuals, educating older adults on disaster preparedness, and providing information about emergency shelter resources (Miyamori et al. 2023; Gibson et al. 2018; Boucher et al. 2022). Caregivers also suggested that organizations like the Alzheimer's Association and Area Agencies on Aging should include training in crisis planning and managing behavioral symptoms during evacuations (Gibson et al. 2018; Schnitker et al. 2019). Caregivers highlighted the importance of involving family members or familiar volunteers in evacuation plans as a strategy to reduce stress (Schnitker et al. 2019).
  System‐level supports, including coordinated emergency planning, infrastructure, and service delivery mechanisms, were also identified as important for effective evacuation. Adequate forecasting of natural disasters could improve shelter setup, ensuring electricity, common medications, and accessible resources outside the hospital (Parks et al. 2021). Caregivers and nursing home managers recommended calm instructions, constant reassurance, and one‐on‐one supervision to prevent persons with dementia from becoming lost during evacuations (Gibson et al. 2018; Schnitker et al. 2019). External memory aids (e.g. maps, instruction cards) and technological devices like tracking tools were proposed to support safety, orientation, and boost the overall functionality of persons with dementia.(Gibson et al. 2018; Schnitker et al. 2019; Woolford et al. 2022; Uekusa 2019). Shared emergency transportation services were also recommended to assist at‐risk individuals (Gibson et al. 2018).
2. *Best practices for non‐evacuation and ongoing support*: In situations where evacuation is not required or feasible, recommendations focused on maintaining continuity of care, access to resources, and caregiver support. Caregivers called for direct support, including hotlines and self‐care resources, highlighting the lack of consideration for their needs in disaster plans despite the heightened stress they experience during emergencies (Boucher et al. 2022; Uekusa 2019).
  Accurate, accessible information tailored to caregivers and persons with dementia was recommended, utilizing methods beyond virtual communication, such as call services or mail (Miyamori et al. 2023; Boucher et al. 2022). Collaboration between care facilities, social workers, local governments, and emergency services was emphasized to support the safety and recovery of persons with dementia (Miyamori et al. 2023; Schnitker et al. 2019). Structural supports, including vulnerable‐person registries were recommended to assist at‐risk individuals (Gibson et al. 2018). Healthcare providers were encouraged to consider environmental factors in patient care, ask targeted questions about behavior during inclement weather, and identify independently living people with dementia who may need extra resources during disasters (Miyamori et al. 2023; Finlay et al. 2020; Yoshida et al. 2021). Establishing food delivery systems for these individuals was also proposed (Miyamori et al. 2023).
  Risks associated with non‐evacuation situations were also highlighted in search and rescue (SAR) contexts. One study noted that the majority of their SAR incidents occurred in temperate (humid) eco‐regions, and that incidents occurring during rain, drizzle, or snow were associated with twofold greater odds of being found dead compared to those during clear weather conditions (Woolford et al. 2022). Among persons with dementia who died following SAR efforts, 97.4% had moderate cognitive impairment, while those with mild or severe dementia represented 2.6% and 0%, respectively (Woolford et al. 2022). The researchers suggest that individuals with moderate cognitive impairment, who may function somewhat independently yet experience confusion, are more prone to becoming lost (Woolford et al. 2022).

## 4 Discussion

Our review highlights three themes regarding the cognitive function of persons with dementia affected by EWEs. Individuals in lower SES conditions appear to experience greater cognitive decline due to home loss and relocation (Hikichi et al. 2020; Shiba et al. 2021; Miyamori et al. 2023; Hikichi et al. 2019; Parks et al. 2021). People from lower SES backgrounds are more likely to live in disaster‐prone areas and face barriers to post‐disaster aid and financial support, making recovery difficult (GovInfo n.d.). Conversely, higher SES and strong social support may serve as protective factors for cognitive decline (W. Zhang et al. 2019), enabling disaster preparedness measures like insurance and home reinforcements (Shiba et al. 2021; Nicosia et al. 2022; Gibson et al. 2018; GovInfo n.d.).

Understanding how EWEs affect preparation and recovery across SES groups may help inform resource allocation strategies aimed at reducing home loss, relocation, and associated cognitive decline (Fussell 2015; Subaiya et al. 2014). Equitable access to financial resources for all disaster‐affected individuals may play an important role in fostering resilience and recovery (GovInfo n.d.; GAO 2022; FEMA 2025). These efforts should extend to pre‐disaster planning including equitable access to preparedness resources, education, and training across diverse socioeconomic, linguistic, and cultural groups. Targeted outreach and culturally responsive education initiatives in lower SES communities may help improve preparedness, reduce risk, and support more effective responses during EWEs.

Research indicates that cognitive function may peak in summer and early fall, influenced by factors like daylight exposure and moderate annual precipitation (20%–40% of days per year) (Finlay et al. 2020; Mooldijk et al. 2021; Khan et al. 2021). Extended periods of indoor confinement during seasonal shifts and EWEs may heighten stress and reduce mental stimulation, mobility and/or physical activity (which are associated with accelerated decline) mirroring effects observed during COVID‐19 lockdowns (Miyamori et al. 2023; Finlay et al. 2020; Yoshida et al. 2021; Kassam and McMillan 2023; Prommas et al. 2023). Digital communication tools (e.g., video calls, social media) and engaging in cognitively stimulating activities (e.g. reading, singing, games) may help persons with dementia maintain social connections and reinforce cognitive skills (Christensen and Castañeda 2014; Kassam and McMillan 2023; Prommas et al. 2023; Hulme et al. 2010; Raglio et al. 2008). Studies should further investigate the short‐ and long‐term efficacy of non‐pharmacological programs like the PLIE program, designed to reduce social isolation, foster mobility, and support resilience in future disasters (Nicosia et al. 2022; Gitlin et al. 2022). Social engagement and community support may play an important role in resilience and cognitive preservation (Hikichi et al. 2020; Shiba et al. 2021; Hikichi et al. 2019; Nicosia et al. 2022; Finlay et al. 2020; Yoshida et al. 2021; Gibson et al. 2018; Huang et al. 2023).

Disaster preparedness and support systems may benefit from being tailored to the needs of people with dementia and their caregivers. Specialized training, enhanced communication, and improved access to resources that facilitate evacuations and recovery efforts may help address barriers identified by caregivers during evacuations (Gibson et al. 2018; Boucher et al. 2022; Schnitker et al. 2019). Caregivers reported significant challenges related to insufficient information on when to evacuate and resources for managing behavioral symptoms of persons with dementia during emergencies (Gibson et al. 2018; Boucher et al. 2022; Uekusa 2019). Programs like Americares, the American Red Cross, FEMA, and the Salvation Army offer online disaster preparation checklists and resource guides (The Salvation Army USA n.d.; Americares n.d.; FEMA 2020; American Red Cross n.d.). Training for healthcare providers is likely important for effective information dissemination. Adults over age 75 represent a rapidly growing group of caregivers, and with rising dementia rates, some caregivers may face dual diagnoses, potentially complicating their ability to care for one another (Y. Zhang et al. 2023; Carr and Utz 2020).

While facilities are required to conduct training and drills for EWEs, one assessment of residential care facility managers indicated that persons with dementia are often excluded from these exercises (Schnitker et al. 2019). Inclusion of persons with dementia in drills may provide long‐term benefits by identifying symptoms exacerbated during disasters and informing improved response strategies. However, such practices may also induce acute distress, which warrants careful ethical consideration. The use of controlled evacuation practices across varying levels of dementia severity (e.g. moderate to high) may generate insights to guide the design of more effective and individualized preparedness strategies. Variations in how dementia severity is defined and classified across studies limit the comparability of these observations. Future research is needed to examine how such drills impact persons with dementia. Technological approaches such as virtual reality simulations, as trialed in cognitive rehabilitation (Tortora et al. 2024; Liao et al. 2020), may also have potential for enhancing training initiatives (Mao et al. 2024; Alshowair et al. 2024). These simulations may help identify behavioral symptoms exhibited by persons with dementia during crises, enhance caregiver preparedness, reduce anxiety, and inform evacuation and support strategies.

While several themes, such as caregiver stress and social isolation, emerged across event types, rapid‐onset events, such as earthquakes, tsunamis, wildfires, and floods, often necessitated immediate evacuation and disruptions to living environments, which were associated with increased behavioral distress, disorientation, and risk of delirium (Miyamori et al. 2023; Parks et al. 2021; Yoshida et al. 2021; Gibson et al. 2018; Boucher et al. 2022; Schnitker et al. 2019; Uekusa 2019). In contrast, slower‐onset environmental exposures, such as precipitation patterns and heatwaves were more frequently associated with gradual changes in cognitive function and required sustained monitoring, environmental adaptation, and ongoing social support (Finlay et al. 2020; Schnitker et al. 2019; Woolford et al. 2022). These findings suggest that adaptive capacity is not uniform across EWE types, but is shaped by the characteristics of the event, as well as geographic, infrastructural, and cultural contexts.

Prior research has identified factors influencing the impact of relocation on persons with dementia in non‐disaster settings (Ryman et al. 2019; Sury et al. 2013). The most harmful scenario to the physical and mental well‐being of persons with dementia appears to be a temporary, involuntary move that disrupts continuity of care and familiarity with the environment (Ryman et al. 2019; Sury et al. 2013). In contrast, relocations that improve prior living conditions are associated with more positive health outcomes, while patient involvement and environmental similarities could help ease transitions (Ryman et al. 2019; Sury et al. 2013). Participation in enrichment and preparation programs at least 6 months before relocation may enhance resilience, though the specifics of these programs are unclear (Y. Zhang et al. 2019; Ryman et al. 2019; Sury et al. 2013). Importantly, the applicability of these findings to disaster‐related relocations remains uncertain and warrants further investigation.

Shelter staff training may be important for addressing caregiver discomfort and hesitancy when evacuating to shelters (Gibson et al. 2018; Boucher et al. 2022). Staff should be equipped to recognize the behavioral symptoms of persons with dementia and implement appropriate privacy practices, parallelling experiences for people with disabilities (Gibson et al. 2018; Boucher et al. 2022). Designated emergency shelters can function as intake centers staffed to assist individuals with disabilities. Familiar staff, furniture, and roommates during relocations have been shown to reduce stress, anxiety, and depression in non‐disaster situations (Ryman et al. 2019; Sury et al. 2013). To mitigate the effects of social isolation, maintaining social cohorts or community structures during relocation may be beneficial where feasible (Hikichi et al. 2020; Nicosia et al. 2022).

### 4.1 Limitations

The small sample size (*n* = 13) limits generalizability, as the available evidence may not fully capture the diversity of experiences across disaster types, care contexts, populations, geographic, and climate regions (Byun et al. 2024). This restricts transferability of findings to other settings with differing socioeconomic, cultural, and disaster‐response infrastructures. For example, findings from the GEJET studies may reflect context‐specific dynamics that are not directly applicable to other regions or climate conditions.

Several articles discussed the participants as a relatively homogenous group of persons with dementia without distinguishing between the types and stages of dementia. Existing literature indicates that planning for EWEs or evacuation responses may vary by the individual's stage of dementia (Christensen and Castañeda 2014). Individuals in the mid‐stages of dementia may demonstrate some awareness and resistance to evacuation, while those in the earlier stages may be more compliant and able to participate in safety preparation (Christensen and Castañeda 2014). Conversely, individuals in the late stages of dementia typically require extensive care and may be unaware of their circumstances, which may allow for smoother evacuations with minimal resistance (Dosa et al. 2023; Christensen and Castañeda 2014).

The categorization of EWEs may oversimplify differences in hazard characteristics. EWEs vary in speed of onset, duration, and associated environmental and logistical challenges (e.g. evacuation or sheltering needs), which may influence both caregiver response and the experiences of persons with dementia. Although this review attempts to account for such variations, the grouping of events may mask distinctions relevant to preparedness and evacuation planning.

The evidence base is further limited by the underrepresentation of contextual factors. Individuals from lower SES backgrounds, for instance, may experience greater cognitive decline following home loss and relocation due to EWEs, but this observation is derived from a single study (Shiba et al. 2021). As such, conclusions regarding SES‐related disparities should be interpreted with caution. Limited geographic diversity highlights the need for larger, more representative samples to better understand how SES influences cognitive outcomes in the context of EWEs and to inform equitable disaster preparedness and resource allocation.

Finally, heterogeneity in study design, outcome measures, and definitions of constructs (e.g. cognitive decline, dementia stage, and adaptive capacity) limits direct comparability across studies. The absence of standardized measures and consistent reporting frameworks introduces challenges for synthesis and may reduce the precision of conclusions drawn from this review. Collectively, these limitations highlight the need for more methodologically consistent, geographically diverse, and conceptually precise research in this area.

### 4.2 Future Directions

Future focus on adaptations for persons with dementia during EWEs should consider different types and stages of dementia to address the various needs across the spectrum of disease (Toots et al. 2016). Tailored intervention strategies for weather event‐specific adaptations should include diverse geographic and demographic populations to understand how cultural, social, and economic factors influence the experiences of persons with dementia and their caregivers during EWEs.

Five major subtypes, including Alzheimer's disease, vascular dementia, Lewy body dementia, and frontotemporal dementia, are associated with distinct cognitive and behavioral phenotypes, with disease progression further determining functional status and care requirements. Integration of these factors is essential for accurate risk stratification and the design of individualized interventions. Such interventions should also include the development of standardized yet flexible toolkits and protocols that can be implemented across settings while remaining responsive to contextual variation and individualizable for care. Emphasis should be placed on practical instructions, including communication strategies, environmental modifications, behavioral support techniques, and decision‐making pathways during evacuation and sheltering scenarios. It is critical to identifying effective environmental and operational supports that promote safety and reduce distress among persons with dementia.

From a geriatric perspective, adaptation strategies should emphasize the principle of minimal resistance, aiming to reduce cognitive burden through simplified communication, minimization of environmental stimuli, and alignment with preserved function. Collaborative care models involving social workers, healthcare providers, and emergency responders should be further explored and operationalized into scalable, replicable frameworks that integrate dementia subtypes and stage into actionable protocols, with incorporation of caregivers and frontline feedback to support iterative refinement.

## 5 Conclusions

This systematic review highlights the challenges faced by persons with dementia during EWEs, focusing on cognitive decline, social isolation, and enhanced disaster preparedness. Community support and socialization are essential in preserving cognitive function and fostering resilience. Improved disaster preparedness requires a framework for promoting climate‐resilient dementia care during EWEs. This should include specialized training for shelter staff, and emergency responders to address the needs of persons with dementia with consideration for cognitive and behavioral phenotypes of dementia. A holistic, collaborative approach that integrates social, technological, and policy‐driven solutions is essential to mitigate the effects of EWEs and promote resilience among persons with dementia in the face of increasingly frequent and intense EWEs.

## Author Contributions

**Sidrah Rafiq**: writing – review and editing, writing – original draft, visualization, validation. **Catherine Chen**: conceptualization, methodology, validation, formal analysis, data curation, writing – original draft, writing – review and editing, visualization, supervision, software, investigation. **Amanda Alvarez**: conceptualization, methodology, writing – original draft, writing – review and editing, formal analysis, data curation, investigation, validation, visualization, software.

## Funding

The authors have nothing to report.

## Ethics Statement

The authors have nothing to report.

## Conflicts of Interest

The authors declare no conflicts of interest.

## Data Availability

The data that support the findings of this study are available from the corresponding author upon reasonable request.
